# Patients’ experience of communication and handling of symptomatic adverse events in breast cancer patients receiving adjuvant chemotherapy

**DOI:** 10.1186/s40900-019-0171-1

**Published:** 2019-11-20

**Authors:** Christina Witt Bæksted, Aase Nissen, Ann S. Knoop, Helle Pappot

**Affiliations:** 1 Documentation & Quality, Danish Cancer Society, Copenhagen, Denmark; 2 Department of Oncology, Rigshospitalet, University Hospital of Copenhagen, Blegdamsvej 9, DK 2100 Copenhagen, Denmark

**Keywords:** Patient-reported experience measures, Patient involvement, Chemotherapy, Breast cancer, Side effects surveillance

## Abstract

**Background:**

The study is based on a national cluster randomized trial investigating the effect of electronic patient-reported outcomes (ePRO) on treatment outcomes in breast cancer patients receiving adjuvant chemotherapy. All 13 oncology departments (11 clusters) treating breast cancer patients in Denmark were randomized to use electronic patient-reported outcomes with real-time clinician feedback (ePRO arm) to track symptoms or usual care for eliciting symptoms using a short paper tracking list (usual care arm). The impact of ePRO on clinical outcomes were examined, which is reported elsewhere. The purpose of the present study was to examine patient-reported experience measure (PREM) regarding communication and handling of side effects/symptoms.

**Methods:**

For this sub-study, patient representatives were involved in the development of a PREM questionnaire. Patients enrolled in the cluster randomized trial completed the PREM questionnaire at their last treatment visit. Semi-structured telephone-interviews were performed with a subgroup of patients. The interviews were based on an interview guide comprised of the questions from the PREM questionnaire and aimed to elaborate on the PREM questionnaire data.

**Results:**

A 12 item PREM questionnaire was developed in partnership with patient representatives. In total, 439 out of 682 patients (64.4%) included patients completed the PREM questionnaire. Telephone semi-structured interviews were performed with 22 patients. In total, 52% (ePRO arm) and 65% (usual care arm) reported having talked with the oncologist/nurse about their responses in the tracking systems before each chemotherapy cycle. Fewer patients in the ePRO arm compared to the usual care arm experienced side effects/symptoms not included in the side effect questionnaire. Patients experienced high satisfaction with oncologists’ and nurses’ handling of side effects/symptoms.

**Conclusions:**

Patients experienced high satisfaction with oncologists’ and nurses’ handling of chemotherapy adverse events. The study indicates a need for a more comprehensive side effect questionnaire as tracking system covering more symptoms than the one used in usual care today.

**Trial registration:**

Clinicaltrials.gov identifier NCT02996201. Registered 19 December 2016, retrospectively registered.

## Plain English summary

This study focuses on breast cancer patients’ experience of communication and handling of symptomatic adverse events, the study contains development of an evaluation questionnaire in partnership with patients. The study is based on a national clinical study in breast cancer patients receiving chemotherapy. The national, clinical study investigated the effect on clinical outcomes of having patients complete electronic questionnaires regarding side effects from treatment on tablet computers before consultation with health professionals. All departments in Denmark treating breast cancer patients were randomly selected to either let patients complete electronic questionnaires on side effects/symptoms or to use standard procedure for eliciting side effects/symptoms. The purpose of the present study was to examine the patients’ experience of communication with health care professionals and the patients’ experience of the handling of side effects/symptoms. To examine the patients’ experience, an evaluation questionnaire was developed in partnership with patient representatives participating in the national, clinical study. All patients in the clinical study were asked to complete the evaluation questionnaire consisting of 12 questions at their last treatment visit. Telephone interviews were performed with a subgroup of patients based on an interview guide, aiming at elaborating on the information gained from the evaluation questionnaire. Out of the 682 patients participating in the clinical study, 439 patients (64.4%) completed the evaluation questionnaire. Patients experienced high satisfaction with the health professionals’ handling of side effects/symptoms. The study indicates a need for a more comprehensive side effect questionnaire covering more symptoms than the one used as standard procedure today.

## Introduction

Surveillance of symptomatic toxicities is an important part of treatment management in cancer patients. Inclusion of patient-reported outcomes (PRO) of symptomatic toxicities has been suggested to provide a more sufficient picture of the patient’s symptomatic adverse events from cancer treatment [1–5] possibly leading to better health-related quality of life in cancer patients and better clinical outcomes [2, 6]. The mechanisms through which PROs are affecting clinical outcomes remain unclear and research within this field is highly needed. PROs have been found to improve symptom awareness in patients and oncologists potentially leading to better medical care of symptoms through better patient-clinician communication [7]. To investigate the potential of PRO, we have performed a national cluster randomized trial in 682 breast cancer patients receiving adjuvant chemotherapy from November 2015 to September 2016 at the 13 oncology departments (11 clusters) in Denmark treating breast cancer [8] (Baeksted CW, Nissen A, Knoop A, Mitchell SA, Christensen J, Johansen C, Pappot H: Routine surveillance for symptomatic toxicities in Danish breast cancer patients— Primary results from a national, cluster randomized trial, submitted). The departments were randomized to either electronic PRO (ePRO) of symptomatic toxicities with real-time clinician feedback or usual care for eliciting symptoms using a short paper tracking list (usual care arm). Electronic PRO was completed at baseline and before each cycle of chemotherapy and was available for the treating oncologist and nurse during consultation with the patient. Based on this randomized trial, the aim of the present study is to describe patients’ experience of communication about and handling of symptomatic toxicities, in the two different situations (ePRO based symptom surveillance and usual care). To describe the patients’ experience properly we found it of importance to include the patients in a part of the research process. Others have after the design of our study demonstrated the benefit of patient involvement in cancer research for example prostate cancer [9]. In our study the involvement of patients in the development of an evaluation questionnaire aimed to make the questionnaire very user-friendly as it was ‘developed with patients for patients’.

## Methods

### Patient involvement in the research process

The cluster randomized trial was funded by a grant from the Danish Cancer Society (R113-A7084-14-S34), who had required user-involvement in the research project as part of the call for funding. A group of researchers all supported by the grant was established to discuss and exchange experiences on patient participation in the research process. In the present study, it was decided to include patient representatives in the development of a Patient-Reported Experiences Measures (PREM) questionnaire.

### PREM questionnaire

The PREM questionnaire was developed in partnership with patients. One patient from each of the participating departments in the national cluster randomized trial was invited to join a group of patient representatives. The only requirements to be included as a patient representative were being enrolled in the cluster randomized trial as a breast cancer patient receiving adjuvant chemotherapy and have access to the internet. At each department, a patient was selected by an oncology nurse at the oncology department. As this was a national study involving patients from all 13 oncology departments across the country, it was decided to use an online platform as communication forum. We used an already existing online platform used for information and communication for cancer patients hosted by the Danish Cancer Society. In this online platform, a closed group was set up in which only the patient representatives and the researcher had access to. Here the researcher could make posts and the patient representatives could communicate with the researcher and each other. Comments from the patient representatives to the posts could be seen by everyone in the group. The researcher contacted each of the patient representatives and explained the task and was available by phone and e-mail during the process for any questions or comments The patient representatives were in the first round asked to suggest questions in four predefined themes: 1) side effects/symptoms, 2) communication with oncologist/nurse about side effects/symptoms, 3) help to alleviate side effects/symptoms, 4) satisfaction with oncologists’/nurses’ handling of side effects/symptoms. These themes were predefined by the researchers and has been found to be associated with the use of PRO in cancer treatment [2, 7, 10, 11]. Based on the patient representatives’ responses, the research team made a draft for a questionnaire that was shared with the patient representatives for comments in the second round. Furthermore, the patients’ understanding of the phrasing of the questions was tested in four other breast cancer patients receiving chemotherapy at Department of Oncology, Rigshospitalet.

### Semi-structured interviews

Telephone semi-structured interviews with a subset of patients completing the PREM questionnaire were aimed to elaborate on the PREM questionnaire data. Each department participating in the cluster randomized trial was asked to select four patients to participate in the interviews. There were no specific inclusion criteria other than acceptance and ability to join a telephone interview with the researcher.

### Data collection and analyses

#### PREM questionnaire

Patients in the cluster randomized trial were asked to complete the PREM questionnaire at their last treatment visit in the oncology departments (February 2016 – September 2016). The PREM questionnaire data was analyzed by comparing distribution of responses for each question for patients from the ePRO arm and usual care arm. The statistical analyses were performed using SAS 9.4. A chi-square test was performed for each of the questions. The tests were considered statistically significant if *p* < 0.05. Data from the patients’ completions of the PREM questionnaire are presented with frequencies and percentages and *p*-values for the chi-square test.

#### Semi-structured interviews

The interviews were performed June–October 2016 immediately after the individual patient had completed the PREM questionnaire and finished the last cycle of chemotherapy. An interview guide including the same questions as the PREM questionnaire was used. The interviews were audio recorded and afterwards transcribed. The interview data were analyzed for content and summarized in themes overall for the two study arms. The interview data are presented with quotations from patients.

## Results

Ten of the 13 invited patient representatives joined the online platform. In the first round, five patient representative provided input and in the second round, five patients commented on the questionnaire draft. Three patients participated in both first and second round, so the unique number of patients participating was seven patients. In cooperation with patient representatives, a 12 items questionnaire covering the four predefined themes and questions on highest attained education, employment status and marital status was developed. The final questionnaire comprised of 10 closed questions with categorical responses and two open-ended questions. In total, 439 patients out of 682 eligible patients (64.4%) included in the cluster randomized trial completed the PREM questionnaire. From the ePRO arm, 55% of patients (191/347) and from the usual care arm, 74% of patients (248/335) completed the PREM questionnaire. The median age was 53 (ePRO arm) and 54 years (usual care arm), respectively. Approximately 10% of patients in each arm had basic school or high school as highest attained education, and 59% (ePRO) and 71% (usual care) were working part/full time (Table 1). In both arms, close to three quarters of patients were married or cohabiting, 75%/78%, respectively (Table 1).

**Table 1:** Characteristics of patients who completed the PREM questionnaire

	ePRO *n* = 191	Usual care *n* = 248
Median age (range) in years	53 (29–76)	54 (22–82)
Highest attained education, n (%)		
Basic or high school	17 (8.9)	33 (13.3)
Vocational education	43 (22.5)	62 (25)
Higher education, 2–4 yrs	110 (57.6)	122 (49.2)
Higher education, ≥5 yrs	19 (9.9)	18 (7.3)
Other	2 (1.0)	11 (4.4)
Missing	0 (0)	2 (0.8)
Employment status, n (%)		
Student	3 (1.6)	3 (1.2)
Working full time	106 (55.5)	107 (43.1)
Working part time	30 (15.7)	39 (15.7)
Unemployed	6 (3.1)	14 (5.6)
Retired	33 (17.3)	59 (23.8)
Other	11 (5.8)	25 (10.1)
Missing	2 (1.0)	1 (0.4)
Marital status, n (%)		
Single	29 (15.2)	22 (8.9)
Married or cohabiting	145 (75.9)	195 (78.6)
Widowed	4 (2.1)	12 (4.8)
Divorced or separated	13 (6.8)	18 (7.3)
Missing	0 (0)	1 (0.4)

Semi-structured telephone interviews were completed with 22 patients (eight from ePRO arm, 14 from usual care arm). The thematic analyses of the semi-structured interviews fell in the four predefined themes: side effects/symptoms of chemotherapy, communication about side effects/symptoms, help to alleviate side effects/symptoms and satisfaction with oncologists’/nurses’ handling of side effects/symptoms.

### Side effects/symptoms

Most patients experienced side effects/symptoms during chemotherapy (96% of patients in ePRO arm and 90% in usual care arm) (Table 2). In the interviews, nausea and fatigue were often mentioned in relation to the first three chemotherapy cycles with Epirubicin/Cyclophosphamide while dry mouth, aching joints and numbness and tingling were frequently pointed out in relation to the last cycles with taxanes (Docetaxel every third week for three cycles or Paclitaxel weekly for 9 weeks).

**Table 2:** Patients’ experience of communication and handling of side effects/symptoms^a^

	ePRO (*n* = 191) n (%)	Usual care (*n* = 248) n (%)	*p* -values×
Did you experience any symptoms/side effects during your treatment with chemotherapy?			*p* = 0.003
Yes	183 (95.8)	222 (89.5)	
No	6 (3.1)	25 (10.1)	
Missing	2 (1.0)	1 (0.4)	
Before each cycle of chemotherapy, you have answered a questionnaire regarding side effects/symptoms. Did you experience any side effects/symptoms not mentioned in the questionnaire?			*p* = 0.005
No	109 (57.1)	95 (38.3)	
Yes	61 (31.9)	113 (45.6)	
Do not know	6 (3.1)	9 (3.6)	
Missing	15 (7.9)	31 (12.5)	
Did you talk with the oncologist/nurse about your answers to the questionnaire regarding side effects/symptoms before each cycle of chemotherapy?			*p* = 0.000
Yes, always	100 (52.4)	160 (64.5)	
Yes, most of the times	38 (19.9)	48 (19.4)	
Yes, some times	35 (18.3)	20 (8.1)	
No, never	12 (6.3)	1 (0.4)	
Do not know	0 (0)	2 (0.8)	
Missing	6 (3.1)	17 (6.9)	
Did you experience any side effects/symptoms between to treatments that you did not talk with the oncologist/nurse about at the following chemotherapy cycle?			*p* = 0.217
No	164 (85.9)	208 (83.9)	
Yes	16 (8.4)	20 (8.1)	
Do not know	4 (2.1)	3 (1.2)	
Missing	7 (3.7)	17 (6.9)	
Do you experience that you have got the help you needed in correlation to alleviating your side effects/symptoms, e.g. medication, physiotherapy, dietitian, counselling?			*p* = 0.225
I did not have any need	15 (7.9)	16 (6.5)	
Yes, to a great extent	157 (82.2)	193 (77.8)	
Yes, to some degree	12 (6.3)	19 (7.7)	
To a lesser extent	1 (0.5)	2 (0.8)	
No, not at all	0 (0.0)	0 (0)	
Do not know	1 (0.5)	0 (0)	
Missing	5 (2.6)	18 (7.3)	
Overall, how satisfied have you been with the way nurses and oncologists have talked with you about/handled your side effects/symptoms?			*p* = 0.066
Very satisfied	163 (85.3)	190 (76.6)	
Satisfied	20 (10.5)	40 (16.1)	
Not satisfied nor unsatisfied	3 (1.6)	2 (0.8)	
Unsatisfied/Very unsatisfied	0 (0)	0 (0)	
Missing	5 (2.6)	16 (6.5)	

^×^*p*-values from chi-square test

^a^Results from PREM questionnaire

The results of the PREM questionnaire showed a high proportion of patients stating that the symptom questionnaire completed in the clinic before each cycle of chemotherapy did not cover all their symptoms. Totally, 32% in the ePRO arm stated that they had experienced symptoms not covered in the questionnaire (electronic PRO questionnaire, including 25 symptoms described by 42 questions) compared to 46% of patients in the usual care arm (paper questionnaire including 10 questions on 10 symptoms) (Table 2).

In the interviews, sleeping problems because of hot flushes and memory problems were mentioned as symptoms not described in the symptom questionnaires. As an example one patient said: *“I think it would be good to tell that in advance (about memory problems, ed.), because I think it will calm people. It’s not because you can do so much about it, but sometimes it is enough to know, that it is normal, and that it will end”* (patient 8, 49 years, ePRO arm).

### Communication with oncologist/nurse about side effects/symptoms

Fifty-two percent of the patients in the ePRO arm reported having talked to the oncologist/nurse before every cycle of chemotherapy about their answers to the questionnaire on symptoms/side effects compared to 65% of patients in the usual care arm (Table 2).

In the interviews, some patients were certain that their answers to the side effect questionnaire was reviewed by a health care professional because the nurse commented on it: *“Well, at least the nurses looked at them. Because they commented on it when I answered differently than last time.”* (Patient 11, age unknown, ePRO arm) while other patients did not have the impression that their answers to the side effect questionnaire were used in the clinic: *“No, I do not think so. They followed their own questionnaire and asked to the answers I gave last time”* (patient 6, 54 years, ePRO arm).

It seemed as if the ePRO questionnaire helped the patients remember their symptoms, one patient said: *“I think that the completion of the side effect questionnaire resulted in “Oh yes, this it was I experienced”, because some time has passed”* (patient 4, age unknown, ePRO arm). Another patient commented: “*I think it made sense to sit and answer and consider, and I had good time and then I have answered and described if there was something additional and then it was registered, and if there was something I was asked about it. I think it is fine, and then I think it saves some time because you don’t have to go through it all with the nurse”* (Patient 5, 51 years, ePRO arm).

To the question on how the conversation on side effect proceeded: *“It’s while you get the chemo. Well, you come in, every third time, you get this tablet computer and answer questions when it is the small treatments (weekly taxanes, ed.) and in three big treatments (Epirubicin/Cyclophosphamide, ed.) there I got it (tablet computer, ed.) every time for completing questions. And when you have started the treatment with chemo, then you sit and talk with the nurse”* (Patient 2, 52 years, ePRO arm) indicating that the conversation on the patient reported symptomatic toxicities happens during treatment with chemotherapy. Some patients stated that they had not talked with the oncologist/nurse about their completion of the side effect questionnaire: *“We did not talk about it, but most of what you answer on the tablet computer is about the symptoms. It was not exactly that she asked: “Well I can see you answered this and this. It was not the way I experienced it was used, but she said at the last time, that she had seen my answers and that it was in accordance with the experience there was (on symptoms, ed.)”* (Patient 2, 52 years, ePRO arm).

In each group, 8% of patients reported having side effects/symptoms not discussed with the oncologist or nurse (Table 2). The most frequent stated reason for this was that patients forgot to tell, and other reasons mentioned was “I did not want to bother”, “I thought it was not important”, “I was not asked”, “I did not want to tell” or “Not enough time” (not shown in Table 2). In the interview patients commented why they had not discussed some symptoms with the oncologist: *“Yes, the blue nails, I had forgot a bit, I hadn’t thought about it..”* (Patient 2, 52 years, ePRO arm).

### Help to alleviate side effects/symptoms

Almost all patients who indicated a need for help from oncologists/nurses to alleviate side effects/symptoms, indicated that they ‘to a great extent’ received the help they needed (91.8% of patients in the ePRO arm and 90.2% in the usual care arm) (Table 2).

### Satisfaction with oncologists’/nurses’ handling of side effects/symptoms

Most patients indicated that they were very satisfied with the oncologists’/nurses’ communication and handling of side effects (85% of patients in ePRO arm and 77% of patients in the usual care arm) (Table 2).

## Discussion

The study showed high satisfaction with communication and handling of symptomatic adverse events among patients in both study arms. A higher proportion of patients in the usual care arm compared to patients in the ePRO arm had experienced side effects that were not described in the side effect questionnaire.

There was a high willingness among patients to participate as patient representatives, however, only about half of the patient representatives participated and gave input. The timing of involving patients in the research process needs to be considered. We asked patients to participate at the time they started their treatment with chemotherapy, and their participation in the research process was simultaneously with their treatment with chemotherapy. This might be a critical point in time as it can be difficult for the patient to take on the task simultaneously with being in cancer treatment.

The PREM questionnaire had to be constructed for the present study, as no validated questionnaire for this purpose was available in Danish language at the time of the study. The fact that patients in general were very satisfied may have resulted in a ceiling effect making it difficult to show a difference between the study arms. The questions in the PREM questionnaire formulated by the patient representative might not have been detailed enough to catch possible points of improvement. For example, more specific questions on how and when the side effects were handled might have revealed possible differences between the two groups. Overall, involvement of the patients has ensured that the questionnaire was meaningful from the patients’ perspective and the response options were adjusted to the patients’ situation and not only health care professionals view on ePRO has been enlighted, but in particular the user-perspective of a user-involving tool. Our choice to develop the PREM questionnaire in partnership with patients enrolled in the study for which the questionnaire was being developed, of course posed a challenge for the time available to develop the questionnaire. The questionnaire had to be developed in the period between the first patients had been enrolled and gained experience with side effects and before this patient finished the treatment (not more than maximum 15 weeks), where the final questionnaire had to be ready for completion. Recently, the Patient Feedback Form developed to be used to evaluate patients’ experience of PRO questionnaires by Snyder et al. was translated and validated in Danish language [12]. The use of this psychometric validated questionnaire may have resulted in a more varied picture and is suggested for future studies. Further, as seen in other studies an additional effect of ePRO may have occurred by measuring health-related quality of life in the two study arms, however this was not the aim of the present study [2].

The most frequent reason mentioned by patients in the PREM questionnaire for not having discussed a symptom with the oncologist or nurse was that the patient had forgotten to tell. This problem could be solved by having patients completing the extended electronic side effect questionnaire continuously at home making it possible to report a symptom as it arises.

Many patients experienced symptoms not addressed in the side effect questionnaire, 32% of patients in the ePRO arm and 46% of patients in the usual care arm. Although, the ePRO questionnaire covers the patient experienced side effect better than the usual care scheme, the results highlight the need for the free text write in field which is only present in the ePRO questionnaire. However, the main focus with using PROs must be to catch side effects which can be handled. The free write-in text field can mainly be used for improved communication, but not for data quantification.

More patients in the usual care arm talked with the oncologist or nurse about the completion of the side effect questionnaire. This might be a consequence of the ePRO being a new system, and it may take time to adjust to it. The difficulties with implementation of ePRO has been described and high-lighted by other investigators [13], and focus must be on PRO tools which are simple to use and reliable. In the future, including review of patients’ completion of ePRO questionnaires in the clinical guidelines might be a solution.

Of the planned 52 interviews, only 22 interviews were completed. The interview data, therefore, represent a small sample of patients completing the PREM questionnaire. Explanation for the fact that only 22 out of the 52 planned interviews were completed might be related to the timing of the interviews. Some patients had agreed to participate in the interviews but declined afterwards. The interviews took place immediately after patients had finished their treatment with chemotherapy, where patients are still affected by the side effects of treatment, especially fatigue can influence ability to perform daily activities at this time-point, which might explain the low participation-rate as patients may need surplus energy to participate in activities like an interview. The completion of the PREM survey also occurred at the time when patients finished their treatment, however, the small number of questions in the PREM questionnaire resulted in higher compliance. Patients might feel that no additional information could be feed-backed to the investigators after already having completed the PREM questionnaire. A higher response rate of the PREM questionnaire was observed in the usual care arm compared to the ePRO arm (74% vs. 55%). This might be influenced by the fact, that the PREM questionnaire together with the patients’ declaration of content was the only extra and the PREM questionnaire was paper based like the symptom questionnaire for tracking in the usual care making it easier to remember for the health care professionals.

## Conclusion

Patients experienced high satisfaction with oncologists’ and nurses’ handling of chemotherapy adverse events. The study indicates a need for a more comprehensive side effect questionnaire for symptom tracking covering more symptoms than the one used in our usual care today.

## Data Availability

The datasets used and/or analyzed during the current study are available from the corresponding author on reasonable request.

## Abbreviations

ePRO: Electronic patient-reported outcomes
PREM: Patient-reported experience measure
PRO: Patient-reported outcomes
